# Risk Factors for Development of Septic Shock in Patients with Urinary Tract Infection

**DOI:** 10.1155/2015/717094

**Published:** 2015-08-25

**Authors:** Chih-Yen Hsiao, Huang-Yu Yang, Chih-Hsiang Chang, Hsing-Lin Lin, Chao-Yi Wu, Meng-Chang Hsiao, Peir-Haur Hung, Su-Hsun Liu, Cheng-Hao Weng, Cheng-Chia Lee, Tzung-Hai Yen, Yung-Chang Chen, Tzu-Chin Wu

**Affiliations:** ^1^Department of Internal Medicine, Ditmanson Medical Foundation Chiayi Christian Hospital, Chiayi, Taiwan; ^2^Department of Hospital and Health Care Administration, Chia Nan University of Pharmacy and Science, Tainan, Taiwan; ^3^Department of Nephrology, Chang Gung Memorial Hospital, College of Medicine, Chang Gung University, Taoyuan, Taiwan; ^4^Department of Emergency, FooYin University Hospital, Pingtung County, Taiwan; ^5^Department of Nurse, Tajen University, Pingtung, Taiwan; ^6^Division of Allergy, Asthma, and Rheumatology, Departmen of Pediatrics, Chang Gung Memorial Hospital, College of Medicine, Chang Gung University, Taoyuan, Taiwan; ^7^Department of Genetics, University of Alabama at Birmingham, Birmingham, AL, USA; ^8^Department of Applied Life Science and Health, Chia Nan University of Pharmacy and Science, Tainan, Taiwan; ^9^Department of Family Medicine, Chang Gung Memorial Hospital, College of Medicine, Chang Gung University, Taoyuan, Taiwan; ^10^Division of Chest, Department of Internal Medicine, Chung Shan Medical University Hospital, Taichung, Taiwan; ^11^School of Medicine, Chung Shan Medical University, Taichung, Taiwan

## Abstract

*Introduction*. Severe sepsis and septic shock are associated with substantial mortality. However, few studies have assessed the risk of septic shock among patients who suffered from urinary tract infection (UTI). *Materials and Methods*. This retrospective study recruited UTI cases from an acute care hospital between January 2006 and October 2012 with prospective data collection. *Results*. Of the 710 participants admitted for UTI, 80 patients (11.3%) had septic shock. The rate of bacteremia is 27.9%; acute kidney injury is 12.7%, and the mortality rate is 0.28%. Multivariable logistic regression analyses indicated that coronary artery disease (CAD) (OR: 2.521, 95% CI: 1.129–5.628, *P* = 0.024), congestive heart failure (CHF) (OR: 4.638, 95% CI: 1.908–11.273, *P* = 0.001), and acute kidney injury (AKI) (OR: 2.992, 95% CI: 1.610–5.561, *P* = 0.001) were independently associated with septic shock in patients admitted with UTI. In addition, congestive heart failure (female, OR: 4.076, 95% CI: 1.355–12.262, *P* = 0.012; male, OR: 5.676, 95% CI: 1.103–29.220, *P* = 0.038, resp.) and AKI (female, OR: 2.995, 95% CI: 1.355–6.621, *P* = 0.007; male, OR: 3.359, 95% CI: 1.158–9.747, *P* = 0.026, resp.) were significantly associated with risk of septic shock in both gender groups. *Conclusion*. This study showed that patients with a medical history of CAD or CHF have a higher risk of shock when admitted for UTI treatment. AKI, a complication of UTI, was also associated with septic shock. Therefore, prompt and aggressive management is recommended for those with higher risks to prevent subsequent treatment failure in UTI patients.

## 1. Introduction

Urinary tract infection (UTI) is one of the leading bacterial infections among adults [1]. It has been estimated that 20–30% of females experience one or more dysuria episodes per year, and most of those episodes represented UTI [2]. The annual incidence of UTI among adults, in addition, was 3% for males and 12.6% for females [3]. Furthermore, the focus of infection among 20–30% of all septic patients had been identified to originate from their urogenital tract, respectively [4].

Severe sepsis and septic shock have been known to associate with substantial mortality and can lead to the consumption of significant amount of health care resources. UTI is characterized by a variety of symptoms ranging from completely asymptomatic to sepsis, severe sepsis, and even septic shock. Although patients with urosepsis have the lowest mortality rate among patients who suffered from all causes of septic shock, urosepsis can still result in a mortality rate as high as 25% to 60% in specific patient groups [5]. Thus, recognition of the risk factors for complications and treatment failure with early intervention of proper broad-spectrum antimicrobials administration may significantly improve the outcome [6, 7].

There are very few studies, however, investigating the risk factors for septic shock among patients with UTI. Therefore, we conducted this study in order to identify the patient groups with higher risk of urosepsis, which may lead to substantial mortality.

## 2. Materials and Methods

### 2.1. Clinical Setting and Subjects

This retrospective study was conducted in Chiayi Christian Hospital, a tertiary referral center located in the southwestern part of Taiwan with a population of 547,000. The hospital is equipped with 1,000 acute care beds with an outpatient department serving approximately 3,800 patients per day and an emergency department serving 260 patients daily. This retrospective observational study complied with the guidelines of the Declaration of Helsinki and was approved by the Medical Ethics Committee. All data were securely protected (by delinking identifying information from the main data sets). Moreover, all primary data were collected according to procedures outlined in STROBE guidelines which strengthen the reporting of observational studies [8].

### 2.2. Definitions and Subjects Assessment

**Figure 1:**
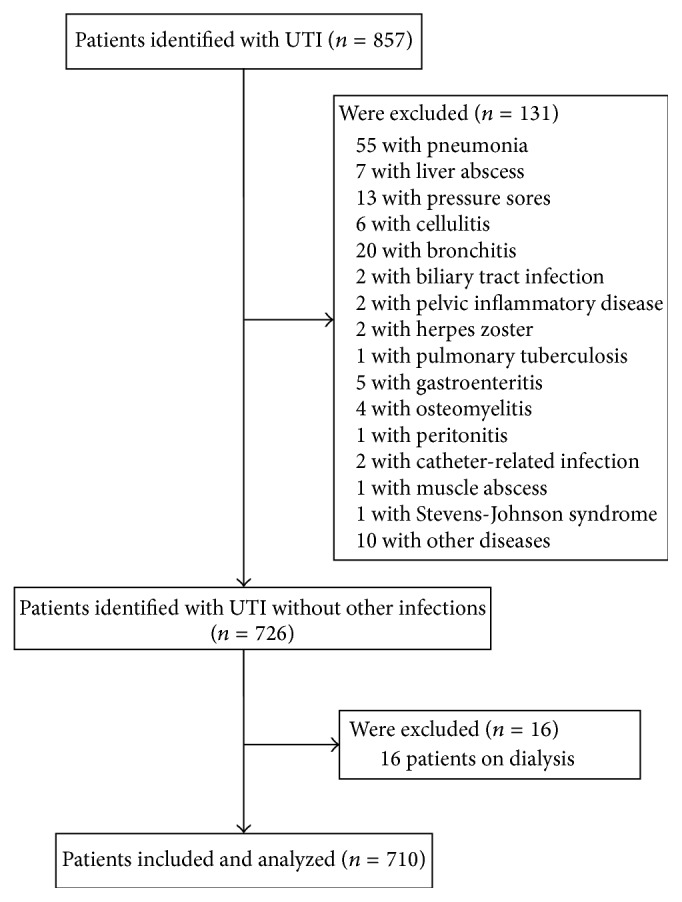
Inclusion and exclusion criteria of our study subjects.

From January 2006 to October 2012, we consecutively studied hospitalized patients with the diagnosis of UTI in Chiayi Christian Hospital. The criteria of UTI in this study are based on clinical symptoms and laboratory diagnosis, including pain on urination (dysuria), lumbago, or fever with bacterial isolation of more than 10^4^ colony forming units (CFU)/mL [9]. Septic shock was defined as sepsis with hypotension (systolic blood pressure (SBP) < 90 mmHg or mean arterial pressure (MAP) < 70 mmHg or SBP decrease > 40 mmHg or less than two standard deviations below normal for age in the absence of other causes of hypotension) over one hour, despite adequate fluid resuscitation at time of admission or during hospitalization [10]. Asymptomatic cases, UTI concurrent with other infection, patients on dialysis therapy, and shocks other than septic shock were excluded, as shown in Figure 1. All data were prospectively collected with a standard form.

### 2.3. Hospital Course

Vital sign measurement, blood sampling, and two or more sets of blood cultures were the standard workups for hospitalized patients with suspected UTI. Comprehensive laboratory data, patient characters, and underlying medical conditions, including age, sex, diabetes mellitus (DM), hypertension, coronary artery disease (CAD), congestive heart failure (CHF), chronic kidney disease (CKD), old cerebrovascular disease (CVA), liver cirrhosis, and cancer, were assessed after admission. Empiric antibiotics were administered within the first hour in all cases suspected with UTI. For hemodynamic stable patients, intravenous first generation cephalosporin plus aminoglycoside (if no impaired renal function) or second generation cephalosporin alone was prescribed initially as empirical antibiotic. For patients with unstable hemodynamic condition, on the other hand, broad-spectrum antibiotics with aggressive intravascular volume replacement were given. Antibiotic adjustment according to the culture results and antimicrobial susceptibility was further arranged during hospitalization. Daily vital signs including blood pressure, temperature, pulse, and respiratory rates were recorded every 8 hours by nurse for the patients who were hemodynamically stable. For the patients who were hemodynamically unstable, vital signs were recorded every 2 to 6 hours a day.

### 2.4. Major Outcomes and Endpoints

The primary outcome was dichotomous. Patients were divided into two groups: (1) cases with septic shock at time of admission or during hospitalization and (2) cases without septic shock. We investigated patient's underlying medical conditions with the potential to contribute to UTI mediated shock. These include (1) underlying general condition (age and gender) and comorbidities (DM, hypertension, CHF, CAD, CKD, liver cirrhosis, old CVA, and malignancy), (2) baseline kidney function, (3) indwelling urinary tract catheter prior to UTI, (4) AKI during hospitalization, and (5) urosepsis. CAD was diagnosed by cardiologist according to resting and exercise electrocardiogram, echocardiography, radionuclide scans, and coronary angiography. CHF was diagnosed according to New York Heart Association Functional Classification. AKI were diagnosed with glomerular filtration rate (GFR) decrease of more than 50% or doubling of serum creatinine during hospitalization compared to baseline renal function according to The* RIFLE criteria*, proposed by the Acute Dialysis Quality Initiative (ADQI) group [11]. GFR was estimated based on serum creatinine and the Modification of Diet in Renal Disease (MDRD) equation. According to the KDOQI CKD classification, the stage of CKD was assigned based on the level of baseline kidney function and irrespective of diagnosis [12]. Bacteremia is an invasion of the bloodstream by bacteria confirmed by blood culture. Comorbidities were obtained through medical chart review and patient interview.

### 2.5. Statistical Analysis

Descriptive statistics were expressed as mean ± standard deviation for continuous variables and percentage for categorical variables. The differences of categorical variables between groups were analyzed by chi-square test, and continuous variables were analyzed by one-way ANOVA test. Multivariate logistic regression analyses were applied to identify risk factors associated with shock during admission. The goodness-of-fit of the logistic regression model was assessed by the Hosmer and Lemeshow test, and the explanatory power was reported with Nagelkerke's pseudo-*R*-square. A two-sided probability value less than or equal to 0.05 was considered statistically significant. Statistical analyses were conducted using SPSS 15.0 for Windows (SPSS Inc., Chicago, IL).

## 3. Results

A total of 710 patients with urinary tract infection (UTI) were enrolled for final analysis. The demographic and clinical characteristics of the enrolled subjects were summarized in Table 1. The mean age of all subjects was 65 ± 19 years old and 484 (68.7%) of them were female. The overall rate of septic shock was 11.3% (80/710), and 23.8% (19/80) of them developed septic shock before hospitalization. The overall mortality rate was 0.28% (2/710). Patients who developed septic shock are older than those who did not (71 ± 16 years old versus 64 ± 19 years old, *P* = 0.001). Higher prevalence of CAD (13.8% versus 5.7%, *P* = 0.006), CHF (12.5% versus 2.5%, *P* < 0.001), and AKI (26.3% versus 11%, *P* < 0.001) and lower baseline eGFR values (66.1 ± 26.9 versus 73.0 ± 27.9 mL/min/1.73 m^2^, *P* = 0.038) were also observed in the septic shock group as compared to those without.

In univariate logistic regression analysis, older age (OR: 1.021, 95% CI: 1.006–1.036, *P* = 0.005), CAD (OR: 2.630, 95% CI: 1.281–5.403, *P* = 0.008), CHF (OR: 5.482, 95% CI: 2.396–12.546, *P* < 0.001), and AKI (OR: 2.894, 95% CI: 1.658–5.052, *P* < 0.001) were independently associated with increased risk of septic shock in patients admitted with UTI. Nonetheless, higher values of baseline eGFR (OR: 0.991, 95% CI: 0.982–1.000, *P* = 0.038) were independently associated with decreased risk of septic shock in UTI patients. As shown in Table 2, in multivariate logistic regression analysis, CAD (OR: 2.521, 95% CI: 1.129–5.628, *P* = 0.024), CHF (OR: 4.638, 95% CI: 1.908–11.273, *P* = 0.001), and AKI (OR: 2.992, 95% CI: 1.610–5.561, *P* = 0.001) were independently associated with increased risk of septic shock, while hypertension (OR: 0.534, 95% CI: 0.304–0.939, *P* = 0.029) was independently associated with decreased risk of septic shock in patients admitted for UTI.

The independent factors for septic shock among female and male UTI patients were listed in Table 3. Congestive heart failure (female, OR: 4.076, 95% CI: 1.355–12.262, *P* = 0.012; male, OR: 5.676, 95% CI: 1.103–29.220, *P* = 0.038, resp.) and AKI (female, OR: 2.995, 95% CI: 1.355–6.621, *P* = 0.007; male, OR: 3.359, 95% CI: 1.158–9.747, *P* = 0.026, resp.) were significantly associated with septic shock in both genders, while DM (OR: 0.491, 95% CI: 0.244–0.988, *P* = 0.046) is independently associated with decrease risk of septic shock in female UTI patients.

## 4. Discussion

Urinary tract infection and septic shock are both among the oldest and most pressing problems in medicine. With increased awareness and advanced modern medicine, clinicians have taken large strides in order to reduce the risk of impending death associated with urosepsis. According to previous studies, the rate of septic shock for UTI patients can range from 20.8% to 32.9% based on different underlying conditions [9, 13, 14]. In the current study, the incidence of septic shock in UTI patients necessitating admission was 11.3%, with a bacteremia rate of 27.9%, AKI 12.7%, and mortality 0.28%. Multiple factors attributing septic shock have previously been reported among UTI patients, including health care-associated infection, liver cirrhosis, and indwelling urinary catheter use [9, 15]. In our study, we have identified that a history of CAD or CHF and AKI are independently associated with septic shock for patients with UTI. We propose that UTI patients with underlying CAD and CHF have increased risk of developing septic shock.

An increase of cardiac burden is noted among patients who suffered from sepsis, particularly severe sepsis. Due to the drop in total peripheral resistance, the effort to maintain adequate blood pressure and mean arterial pressure has to include an increase of cardiac output (CO). In addition, retention of large numbers of leukocytes in the coronary microcirculation [16], autonomic dysfunction, inflammation-induced intrinsic myocardial depression [17], mitochondrial dysfunction, and apoptosis [18] can all contribute to the development of sepsis-induced myocardial dysfunction and lead to depressed cardiac contractility in sepsis patients. With transesophageal echocardiography, Vileillard-Baron et al. found that 60% of intubated patients with septic shock experienced global left ventricular (LV) hypokinesis during the first 3 days of sepsis [19]. Three important cardiovascular events have been identified as sepsis progresses from severe sepsis to septic shock. This includes a reduction in intravascular volume due to capillary leak, a decrease of vascular tone, and a suppressed cardiac contractility [20]. UTI patients with severe sepsis may present with both LV ejection fraction (EF) depression and severe stroke volume reduction. When CO is unable to increase appropriately for compensation, the drop in mean arterial pressure leads to septic shock. Patients with myocardial dysfunction have significantly higher mortality rate when compared with septic patients without cardiovascular impairment [21]. However, to our knowledge, there is no previous study which investigated the risk of myocardial dysfunction in UTI patients. In our study, the percentages of CAD and CHF among the subjects with septic shock were 13.8% and 12.5%, whereas they were 5.7% and 2.5% among the subjects without septic shock. The odds ratios for septic shock among patients with CAD and CHF were 3.78 and 4.64, respectively. Patients with CAD or CHF are more likely to have an impaired cardiac contractility and myocardial dysfunction. Septic shock, additionally, can lead to further cardiac contractility deterioration in those unable to increase cardiac output appropriately. Therefore, patients with CAD or CHF along with severe UTI are more likely to progress to septic shock than those without. To prevent imminent mortality in UTI patients with underlying CAD and CHF, vigilance fluid resuscitation and early broad-spectrum antimicrobial therapy may be recommended.

Older age is also associated with higher risk of septic shock in UTI patients (71 ± 16 versus 64 ± 19 years old, *P* = 0.001) with an odds ratio of 1.021 incremented each year in univariate logistic regression model. The prevalence of UTI in the elderly is much higher than that in younger individuals. As reported, at least 20% of women and 10% of men aged 65 years or older have bacteriuria [22]. In our study, 59.8% (423/710) of all cases are over the age of 65. Various pathogen defensing mechanisms have been known to change along with age. These include recession of cell-mediated immunity, obstructive uropathy and neurogenic dysfunction related bladder defense alteration, bacterial receptivity intensification of uroepithelial cells [23], fecal and urinary incontinence related contamination, urethral instrumentation and catheterization, and hormone related antibacterial factors reduction in the prostate and vagina [24]. Nonetheless, age is a long known traditional risk factor for hypertension, dyslipidemia, impaired glucose tolerance, and obesity. Since these remain the major modifiable risk factors for the development of coronary artery disease afflicting the elderly [25], it is reasonable to assume that, aside from host immune alteration, CAD may also contribute to the progression of urosepsis to septic shock among the elderly. In our study, older patients (aged 65 years or older) had a higher rate of CAD (9.69% (41/423) versus 2.09% (6/287)) and CHF (5.44% (23/423) versus 1.05% (3/287)) compared to younger patients. Moreover, all patients (11/11) with CAD and UTI related septic shock and 90% (9/10) of the patients with CHF and septic shock were aged 65 years or more, respectively. The elderly are definitely in risk for UTI related septic shock in our analysis. We hypothesized that this may be due to defense mechanism modification or the development of cardiac diseases such as CHF and CAD.

Sepsis and septic shock are the most common triggers of AKI [26]. Yet, the pathophysiology of AKI in sepsis is complex and multifactorial. This includes intrarenal hemodynamic changes, endothelial dysfunction, infiltration of inflammatory cells in the renal parenchyma, intraglomerular thrombosis, and obstruction of tubules with necrotic cells and debris [27]. Because septic shock can further activate sympathetic nervous system and the renin-angiotensin-aldosterone axis, patients with septic shock are more likely to have AKI [28]. Previous studies have suggested that septic shock can contribute to the development of AKI up to 45 to 60% [29, 30], and patients with severe AKI have increased risk of end-stage renal disease and even death. In our study, we found that patients with septic shock had higher chance of AKI compared to those without. Specifically, 26.3% of UTI patients with septic shock developed AKI episode. Since sepsis-related AKI leads to poor outcomes, early and appropriate antibiotic therapy, aggressive resuscitation, and close monitoring of changes in renal function are necessary to early detect and to prevent the development of AKI in patients with urosepsis.

Due to anatomic differences, the complexity of UTI among males and females is considerably different. However, little is known about the risk of septic shock between the two. Herein, we showed that both genders shared similar risk factors. CHF is significantly a risk factor for septic shock and AKI is associated with septic shock in both genders. Though the incidence of UTI may vary between genders, the risk of comorbidity for UTI patients remains similar between the two.

There are several limitations in our study. First, the data collection in retrospective studies may be biased and affected by missing data. However, in attempt to avoid bias, we prospectively collected the data with a standard form. In addition, the process was supervised by a senior nephrologist through weekly meetings. Second, the choices of empirical antimicrobial agents were made by attending physicians without strict unification. However, the prescription of antibiotics was supervised by the infection control unit in the hospital with protocols specifying the use of antibiotics. As a result, the variations of antibiotics used between different physicians were not profound in this study. Third, our study was done in a single institute and hence the results may not be generalized. However, because our hospital is an acute care hospital, the patients in this study were not highly selected. This reduces the limiting generalizability of the results.

In conclusion, we proclaimed that patients with a medical history of CAD or CHF have higher risk of septic shock when suffering from UTI despite treatment. Additionally, patients with septic shock related to UTI had a risk of developing AKI. Therefore, early and aggressive management is recommended for UTI patients, especially those in the high risk group, to prevent subsequent treatment failure in UTI patients.

## Figures and Tables

**Table 1:** Characteristics of the 710 patients with urinary tract infection.

Characteristics	All (*n* = 710)	Presence of septic shock		*P* value
		Yes	No	
		(*n* = 80)	(*n* = 630)	
Age (year)	65 ± 19	71 ± 16	64 ± 19	0.001
Gender				
Male	226 (31.8%)	30 (37.5%)	196 (31.1%)	0.248
Female	484 (68.2%)	50 (62.5%)	434 (68.9%)	
Diabetes mellitus	271 (38.2%)	26 (32.5%)	245 (38.9%)	0.268
Hypertension	285 (40.1%)	30 (37.5%)	255 (40.5%)	0.609
Coronary artery disease	47 (6.6%)	11 (13.8%)	36 (5.7%)	0.006
Congestive heart failure	26 (3.7%)	10 (12.5%)	16 (2.5%)	<0.001
Liver cirrhosis	33 (4.6%)	4 (5.0%)	29 (4.6%)	0.780
Malignancy	76 (10.7%)	8 (10.0%)	68 (10.8%)	0.829
Old cerebrovascular accident	158 (22.3%)	24 (30.0%)	134 (21.3%)	0.077
Indwelling Foley catheter	45 (6.3%)	8 (10.0%)	37 (5.9%)	0.154
Bacteremia	198 (27.9%)	29 (36.3%)	169 (26.8%)	0.077
Acute kidney injury	90 (12.7%)	21 (26.3%)	69 (11.0%)	<0.001
Baseline eGFR (mL/min/1.73 m^2^)	72.2 ± 27.9	66.1 ± 26.9	73.0 ± 27.9	0.038
Chronic kidney disease stage				
1	182 (25.6%)	11 (13.8%)	171 (27.1%)	0.048
2	284 (40.0%)	33 (41.3%)	251 (39.8%)	
3	201 (28.3%)	32 (40.0%)	169 (26.8%)	
4	30 (4.2%)	3 (3.8%)	27 (4.3%)	
5	13 (1.8%)	1 (1.3%)	12 (1.9%)	

Data are expressed as mean ± SD or number (percentage).

**Table 2:** Logistic regression model for factors related to septic shock (*n* = 710).

	Univariate			Multivariate		
	OR (95% CI)		*P* value	OR (95% CI)		*P* value
Age (year)	1.021	(1.006–1.036)	0.005	1.014	(0.996–1.033)	0.129
Gender (female)	0.753	(0.464–1.220)	0.249	1.059	(0.627–1.787)	0.831
Diabetes mellitus	0.757	(0.461–1.241)	0.269	0.629	(0.364–1.085)	0.096
Hypertension	0.882	(0.546–1.426)	0.609	0.534	(0.304–0.939)	0.029
Coronary artery disease	2.630	(1.281–5.403)	0.008	2.521	(1.129–5.628)	0.024
Congestive heart failure	5.482	(2.396–12.546)	<0.001	4.638	(1.908–11.273)	0.001
Liver cirrhosis	1.091	(0.373–3.187)	0.874	1.109	(0.357–3.443)	0.858
Malignancy	0.918	(0.424–1.988)	0.829	0.654	(0.282–1.513)	0.321
Old cerebrovascular accident	1.586	(0.948–2.655)	0.079	1.711	(0.933–3.139)	0.083
Indwelling Foley catheter	1.781	(0.798–3.973)	0.159	1.490	(0.631–3.521)	0.363
Bacteremia	1.551	(0.951–2.529)	0.078	1.610	(0.958–2.707)	0.072
Acute kidney injury	2.894	(1.658–5.052)	<0.001	2.992	(1.610–5.561)	0.001
Baseline eGFR (mL/min/1.73 m^2^)	0.991	(0.982–1.000)	0.038	0.997	(0.986–1.008)	0.602

**Table 3:** Multivariate logistic regression model for factors related to septic shock by gender.

	Female			Male		
	OR (95% CI)		*P* value	OR (95% CI)		*P* value
Age (year)	1.011	(0.988–1.035)	0.345	1.023	(0.988–1.059)	0.199
Diabetes mellitus	0.491	(0.244–0.988)	0.046	0.972	(0.389–2.434)	0.952
Hypertension	0.486	(0.233–1.013)	0.054	0.626	(0.247–1.588)	0.324
Coronary artery disease	2.626	(0.928–7.428)	0.069	2.236	(0.570–8.772)	0.249
Congestive heart failure	4.076	(1.355–12.262)	0.012	5.676	(1.103–29.220)	0.038
Liver cirrhosis	1.541	(0.395–6.006)	0.533	0.642	(0.071–5.774)	0.693
Malignancy	0.908	(0.333–2.475)	0.851	0.276	(0.053–1.434)	0.126
Old cerebrovascular accident	1.928	(0.832–4.467)	0.126	1.624	(0.638–4.134)	0.309
Indwelling Foley catheter	1.034	(0.258–4.144)	0.963	2.467	(0.758–8.024)	0.133
Bacteremia	1.467	(0.745–2.888)	0.268	1.887	(0.793–4.490)	0.151
Acute kidney injury	2.995	(1.355–6.621)	0.007	3.359	(1.158–9.747)	0.026
Baseline eGFR (mL/min/1.73 m^2^)	0.994	(0.980–1.010)	0.470	0.997	(0.981–1.014)	0.762
